# Integrated Bulk and Single‐Cell Transcriptomic Analysis Reveals Xenobiotic Metabolism Genes Drive Progression From Liver Cirrhosis to Hepatocellular Carcinoma

**DOI:** 10.1155/humu/6845605

**Published:** 2026-05-13

**Authors:** Hao Xu, Yanpeng Li, Rui Zhao, Shaowei Ma, Ning Hu, Shuo Yu, Xuefeng Lv

**Affiliations:** ^1^ Department of Laboratory Medicine, The Third Affiliated Hospital of Zhengzhou University, Zhengzhou, Henan, China, zdsfy.net; ^2^ Zhengzhou Key Laboratory for In Vitro Diagnosis of Hypertensive Disorders of Pregnancy, Zhengzhou, Henan, China; ^3^ Department of Emergency, Hebei General Hospital, Shijiazhuang, Hebei, China, hebmu.edu.cn; ^4^ Department of Gastrointestinal Surgery, The Second Hospital of Hebei Medical University, Shijiazhuang, Hebei, China, hebmu.edu.cn; ^5^ Department of Surgical Oncology, The Second Hospital of Hebei Medical University, Shijiazhuang, Hebei, China, hebmu.edu.cn

**Keywords:** hepatocellular carcinoma, liver cirrhosis, machine leaning, single-cell RNA-seq, xenobiotic metabolism

## Abstract

**Background:**

Hepatocellular carcinoma (HCC) is one of the most prevalent malignant tumors globally, with liver cirrhosis (LC) recognized as a significant precursor. Xenobiotic metabolism plays a pivotal role in liver diseases, where the liver′s primary function as a detoxifying organ directly influences health and tumor development. Therefore, exploring the function of genes associated with xenobiotic metabolism in patients with HCC and LC is crucial for advancing diagnosis and treatment strategies.

**Methods:**

This study integrated bulk transcriptome RNA sequencing and single‐cell RNA sequencing data of HCC and LC from the GEO database. Differential expression analysis, GO and DO enrichment analyses, and PPI network construction were performed to identify hub genes. ROC curve analysis was used to assess the diagnostic value of these genes, and potential therapeutic drugs were predicted using drug databases. The expression of the identified hub genes was validated in clinical tissue samples (≥ 10 pairs) by qRT‐PCR and Western blot. Functional assays, including wound healing, transwell migration, and invasion assays, were conducted following siRNA‐mediated knockdown of selected hub genes in Huh7 hepatoma cells.

**Results:**

We identified five key xenobiotic metabolism‐related hub genes: AKR1C3, CYB5A, ADH1C, MAOA, and ALDH2. These genes were significantly overexpressed in HCC and LC tissues compared with normal tissues and were associated with xenobiotic metabolism, substance abuse, alcohol use disorders, and cancer. ROC curve analysis indicated that these hub genes have high diagnostic value in HCC and LC. Potential drug prediction identified six compounds, including retinal, isopropanol, and disulfiram, which may have therapeutic effects. Clinical tissue validation confirmed that all five hub genes were significantly upregulated at both mRNA and protein levels in HCC and LC tissues compared with normal controls (*p* < 0.05). Functional experiments demonstrated that knockdown of ALDH2 or AKR1C3 significantly impaired the migration and invasion abilities of Huh7 cells, with a reduction of over 50% in transwell assays (*p* < 0.05).

**Conclusion:**

This study systematically analyzed the expression characteristics and functional significance of xenobiotic metabolism‐related genes in HCC and LC by integrating multiple high‐throughput sequencing technologies. Experimental validation confirmed the upregulation of these hub genes in clinical tissues and demonstrated that ALDH2 and AKR1C3 promote hepatoma cell migration and invasion, providing experimental evidence for their potential roles in disease progression. Potential drugs targeting these hub genes were preliminarily investigated.

## 1. Introduction

Hepatocellular carcinoma (HCC) is one of the most common malignant tumors worldwide, characterized by high incidence and mortality rates [1]. The pathogenesis of HCC is complex, involving various genetic mutations and dysregulated signaling pathways [2, 3]. Liver cirrhosis (LC) is a significant precursor to HCC, with approximately 80%–90% of HCC patients having an underlying cirrhotic condition [4, 5]. Therefore, understanding the molecular links between HCC and LC is crucial for early diagnosis and treatment.

Xenobiotic metabolism refers to the metabolic processes of exogenous chemical substances, including drugs, environmental pollutants, and food additives [6, 7]. These xenobiotic compounds undergo biotransformation primarily through the liver′s metabolic enzyme system, ultimately being excreted from the body [8, 9]. As the central detoxification organ, the liver plays a pivotal role in xenobiotic metabolism [10]. Abnormalities in these metabolic pathways can lead to liver cell damage and carcinogenesis, closely associated with the development and progression of LC and HCC [11, 12].

With the advancement of high‐throughput sequencing technologies, integrating large‐scale bulk RNA‐seq and single‐cell RNA‐seq data have provided new insights into the complex heterogeneity of the tumor microenvironment. This integrative analysis approach not only reveals differences between tumor and normal tissues but also delves into the functional roles of different cell types in xenobiotic metabolism [13]. This is crucial for understanding the molecular mechanisms of HCC and identifying new prognostic markers.

Although several studies have examined xenobiotic metabolism genes in liver cancer or fibrosis individually [14, 15], few have simultaneously characterized these genes across both HCC and LC using integrated bulk and single‐cell transcriptomic data. This integrated approach is crucial because HCC predominantly arises from a background of LC, creating a clinical and biological continuum. Analyzing both conditions together allows us to identify molecular alterations that persist from premalignant stages into overt cancer, potentially revealing key drivers of tumor initiation and progression. Our study not only evaluates these genes from a prognostic perspective across multiple cohorts but also explores their relevance to immune infiltration and therapeutic repurposing. In this study, we perform a comprehensive analysis.

## 2. Methods

### 2.1. Data Preprocessing

HCC and LC bulk transcriptome RNA‐seq and scRNA‐seq data were retrieved from the GEO database (https://www.ncbi.nlm.nih.gov/geo/), including GSE146115, GSE76247, GSE136103, and GSE14323. All samples from these datasets contained complete clinical and survival information; therefore, no samples were excluded due to missing data (*n* = 0 excluded). The “limma” and “sva” R packages were utilized for background correction, quantile normalization, and batch effect removal. All bulk transcriptome data were normalized based on the log2‐converted FPKM (fragments per kilobase of transcript per million mapped reads) values for sequent analysis.

### 2.2. scRNA‐Seq Analysis

All scRNA‐seq data were analyzed in conjunction with the “Seurat” and “SingleR” R packages, including filtering, normalization, and unsupervised clustering. Similarly, mitochondrial counts over 5% and cells with unique feature counts below 200 or over 10,000 were eliminated from the analysis. A normalization operation was done on the filtered matrix using Seurat′s NormalizeData function. The obtained matrix was converted into a Seurat object using the “Seurat” R package and integrated using “Harmony” R package. To address potential batch effects between the GSE146115 and GSE136103 datasets, we applied the Harmony algorithm (v1.0) on the Top 30 principal components derived from the merged Seurat object. Harmony‐corrected embeddings were then used for subsequent dimensionality reduction (UMAP), clustering, and downstream analyses. All procedures followed the standard pipeline recommended by the Harmony developers [16].

UMAP was generated from the principal component analysis (PCA) output using “RunUMAP” function. Clustering analysis was carried out using “FindClusters” function with Top 15 principal components and a resolution of 0.5. The “FindAllMarkers” function was performed to screen differentially expressed genes (DEGs) of each cluster within the criteria of log2 fold change (FC) > 0.5 and adjusted *p* < 0.05. “SingleR” R package was conducted for cell‐type annotation.

### 2.3. Identification of Hallmark Genes

DEGs for HCC and LC were identified from the bulk transcriptome RNA‐seq data by comparing tumor/cirrhosis samples versus normal samples using the “limma” R package. Genes with |log2 FC| > 1 and adjusted *p* value of < 0.05 were considered statistically significant DEGs and were defined as “HCC marker genes” and “LC marker genes” in the context of this study. The hallmark genes related to xenobiotic metabolism were obtained from the MSigDB database (https://www.gsea-msigdb.org/gsea/msigdb, HALLMARK_XENOBIOTIC_METABOLISM). An intersection among xenobiotic metabolism hallmark genes, HCC DEGs, and LC DEGs was mapped using the Venn diagram tool (https://bioinformatics.psb.ugent.be/webtools/Venn/). The “limma” R package was used to analyze differential expression levels of the intersection genes, which were visualized by heatmap.

### 2.4. Function Enrichment Analysis

Gene ontology (GO) and disease ontology (DO) enrichment analyses were performed for each candidate gene using “ClusterProfiler” R package to determine the functional roles and related pathways. Visualization of the results was performed using R packages “circlize” and “GOplot” R package.

### 2.5. Analysis of Protein–Protein Interaction (PPI)

PPI network was produced by STRING (http://string-db.org/) based on uploading the intersection genes. It provides a critical assessment and integration of direct and indirect (physical) PPIs. Genes with a combined significance score above 0.7 were deemed as hubs. “Limma” R package was performed for acquiring differential expression levels of hub genes. Receiver operating characteristic (ROC) curve of these hub genes was produced by the “pROC” R package.

### 2.6. Correlation Analysis of Hub Genes With Immue Cells

The expression correlation diagram was drawn using the “corrplot” R package. The CIBERSORT algorithm was used to estimate the relative proportions of 22 immune cell types based on the gene expression profiles. To assess the correlation between hub gene expression and immune cell fractions, Pearson correlation coefficients were calculated. To account for multiple testing and control for false positives, *p* values were adjusted using the Benjamini–Hochberg false discovery rate (FDR) method. Correlations with an adjusted *p* value of < 0.05 were considered statistically significant.

### 2.7. Protential Drug Prediction

Drug Signatures database (DSigDB, http://tanlab.ucdenver.edu/dsigdb), an online database of unique compounds and corresponding genes, holds thousands of gene sets. DSigDB was utilized to predict protein–drug interactions and identify potential pharmacological compounds for the hub genes, which benefit HCC or LC. PubChem (https://pubchem.ncbi.nlm.nih.gov) provided SDF versions of the two‐dimensional structural data of potential drugs.

### 2.8. mRNA Purification and Reverse Transcription Quantitative PCR (RT‐qPCR)

Total RNA was isolated from clinical specimens using TRIzol reagent (Sigma‐Aldrich, Cat. No. T9424, United States) following the manufacturer′s protocol. First‐strand complementary DNA (cDNA) was synthesized from total RNA with the HiFi II 1st Strand cDNA Synthesis Kit (Yeasen, Cat. No. 11119ES60, China). Quantitative real‐time PCR (qPCR) was performed using SYBR Green Master Mix (Yeasen, Cat. No. 11184ES03, China) on an Applied Biosystems real‐time PCR system. Relative mRNA expression levels were determined using the 2^−*Δ* *Δ* *C* *t*^ method and normalized to GAPDH. All samples were analyzed in triplicate. Primer sequences are listed in Table S1.

### 2.9. Western Blot Analysis

Tissues were lysed in RIPA buffer supplemented with protease and phosphatase inhibitors. Protein concentrations were determined using a BCA protein assay kit. Equal amounts of protein (20 *μ*g) were separated by SDS–PAGE and transferred onto NC membranes.

Membranes were blocked with 5% nonfat milk in TBST for 1 h at room temperature and then incubated with primary antibodies overnight at 4°C. The following primary antibodies were used: Anti‐AKR1C3 Polyclonal Antibody (Proteintech, Cat. No. 84872‐5‐RR, United States); anti‐ADH1C polyclonal antibody (Proteintech, Cat. No. 18897‐1‐AP, United States); anti‐CYB5A polyclonal antibody (Proteintech, Cat. No. 12365‐1‐AP, Unites States); anti‐ALDH2 polyclonal antibody (Proteintech, Cat. No. 15310‐1‐AP, United States); anti‐MAOA polyclonal antibody (Proteintech, Cat. No. 10539‐1‐AP, United States); and anti‐alpha tubulin polyclonal antibody (Proteintech, Cat. No. 80762‐1‐RR, United States). After washing with TBST, membranes were incubated with HRP‐conjugated secondary antibodies for 1 h at room temperature. Protein bands were visualized using an enhanced chemiluminescence (ECL) detection system and quantified by ImageJ software. Alpha‐tubulin was used as the loading control.

### 2.10. RNA Interference (RNAi)

For transient knockdown of AKR1C3 or ALDH2, cells were transfected with a pool of three gene‐specific siRNAs targeting AKR1C3 (sense sequences: 5 ^′^‐CCUGAAGAAGUGGAAGAUA‐3 ^′^, 5 ^′^‐GCAUGAAGAUGGAGAAGAU‐3 ^′^, and 5 ^′^‐GAAGCUGCAUUGACUACAAU‐3 ^′^) or ALDH2 (sense sequences: 5 ^′^‐GCAUGAAGGAACUGAAGAA‐3 ^′^, 5 ^′^‐CCGCAGCUGUCUUCACAAA‐3 ^′^, and 5 ^′^‐GGAGAAGCUUACCAACAU A‐3 ^′^). A nontargeting siRNA was used as a negative control. Transfections were performed using X‐tremeGENE siRNA Transfection Reagent (Roche, Cat. No. 4476115001, Switzerland) following the manufacturer′s protocol.

### 2.11. Wound‐Healing Assay

The transfected cells were cultured in a 12‐well plate. Upon reaching confluence, a pipette tip was used to introduce a vertical scratch, followed by the replacement of the culture medium with a serum‐free medium. The initial scratch width was recorded, and the healed scratch width was measured after a specified time interval.

### 2.12. Transwell Migration and Invasion Assays, and Transendothelial Migration (TEM) Models

For the migration assay, transfected cells (Huh7: 4 × 10^4^/well) were resuspended in a serum‐free medium and seeded into the upper chamber of a transwell system (Corning, Cat. No. 3422, United States). A complete medium containing 20% FBS was added to the lower chamber. After a specific incubation period (24 h), cells that had migrated through the membrane and adhered to the underside were fixed with 4% paraformaldehyde for 20 min, stained with crystal violet for 25 min, and counted under an IX73 fluorescence microscope. For the invasion assay, the lower chamber was precoated with Matrigel (Corning, Cat. No. 354248, United States), and all other procedures were identical to the migration assay.

### 2.13. Statistical Analysis

Data analysis was performed using the R software (Version 4.2.2). Wilcoxon signature rank sum test was used for paired comparison, and Kruskal rank sum test was used for multigroup comparison. The correlation was calculated by Pearson method. Statistical significance was defined as *p* < 0.05.

## 3. Results

### 3.1. ScRNA‐Seq Analysis of HCC and LC Samples

**Figure 1:**
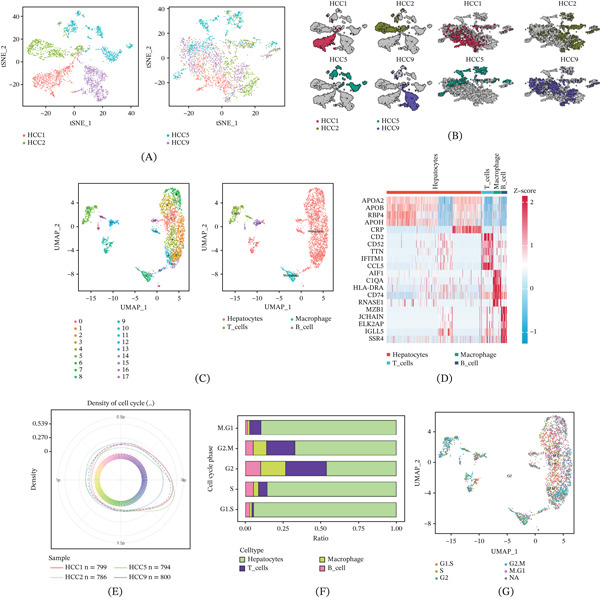
ScRNA‐seq analysis of four HCC samples. (A–B) t‐SNE plot from four primary HCC samples before and after batch effect removal. (C) UMAP of the distribution of 18 cell clusters. (D) Heatmap of the expressions of Top 20 marker genes in 18 cell clusters. (E) Density plot of different cell cycle phases of each cell type. (F) Bar chart of distribution of cell cycle phases in each cell type. (G) UMAP plot of cell cycle phases of single cells.

Gene expression profiles of a total of 3200 cells from 4 HCC samples were retrieved from GSE146115 and analyzed in Figure 1A–B. Eighteen distinct clusters were identified after UMAP analysis. As shown in Figure 1C, Cluster 7 corresponded to macrophage cells, Clusters 10 and 16 corresponded to B cells, Clusters 5 and 15 corresponded to T cells, and other clusters corresponded to hepatocytes. Expressions of the Top 20 marker genes in different cell clusters are shown in Figure 1D, including APOA2, APOB, RBP4, APOH, CRP, CD2, CD52, TTN, IFITM1, CCL5, AIF1, CIQA, HLA‐DRA, CD74, RNASE1, MZB1, JCHAIN, ELK2AP, IGLL5, and SSR4. The percentages of cells within each cell cycle phase are depicted by density plots (Figure 1E). The distribution of cell cycle phases of each cell type in Figure 1F shows that immune cells like T cells, B cells, and macrophages mainly focus on G2 compared with other phases. UMAP plot (Figure 1G) indicates the transcriptional cell cycle identity per cell.

**Figure 2:**
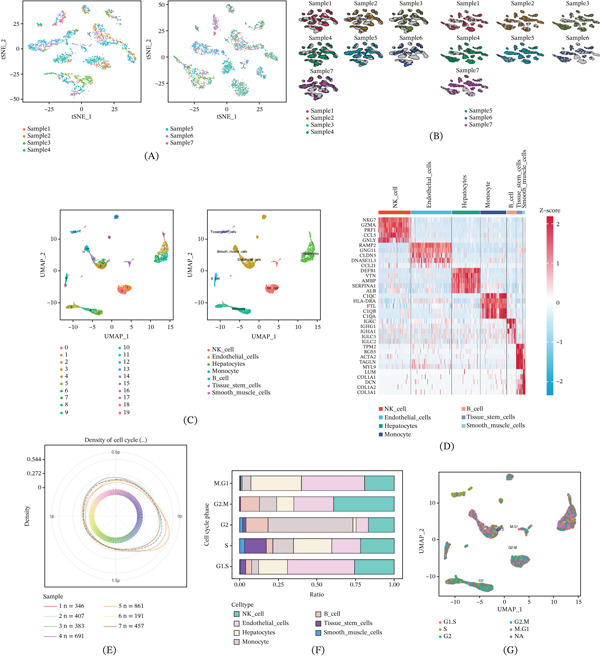
ScRNA‐seq analysis of seven LC samples. (A–B) t‐SNE plot from seven primary LC samples before and after batch effect removal. (C) UMAP of the distribution of 20 cell clusters. (D) Heatmap of the expressions of Top 35 marker genes in 20 cell clusters. (E) Density plot of different cell cycle phases of each cell type. (F) Bar chart of distribution of cell cycle phases in each cell type. (G) UMAP plot of cell cycle phases of single cells.

Similarly, gene expression profiles from seven LC samples were obtained from GSE136103 and analyzed in Figure 2A–B. Twenty distinct clusters were identified after UMAP analysis. As shown in Figure 2C, Clusters 9 and 10 correspond to B cells, Clusters 0 and 1 correspond to NK cells, Cluster 4, 5, and 8 correspond to monocytesC cluster 12 corresponds to stem cells, and Cluster 3, 7, 11, and 17 correspond to hepatocytes; other clusters correspond to smooth muscle cells and endothelial cells. Expressions of Top 35 marker genes in various cell clusters are exhibited in Figure 2D, including NKG7, GZMA, PRF1, CCL5, GNLY, RAMP2, GNG11, CLDN5, DNASE1L3, CCL21, DEFB1, VTN, AMBP, SERPINA1, ALB, C1QC, HLA‐DRA, FTL, C1QB, C1QA, IGKC, IGHG1, IGHA1, IGLC3, IGLC2, TPM2, RGS5, ACTA2, TAGLN, MYL9, LUM, COL1A1, DCN, COL1A2, and COL3A1. The percentages of cells within each cell cycle phase are depicted by density plots (Figure 2E). The distribution of cell cycle phases of each cell type in Figure 2F shows that immune cells like NK cells, B cells, and macrophages mainly focus on G2 compared with other phases. UMAP plot (Figure 2G) indicates the transcriptional cell cycle identity per cell.

### 3.2. Identification of Hallmark Genes

**Figure 3:**
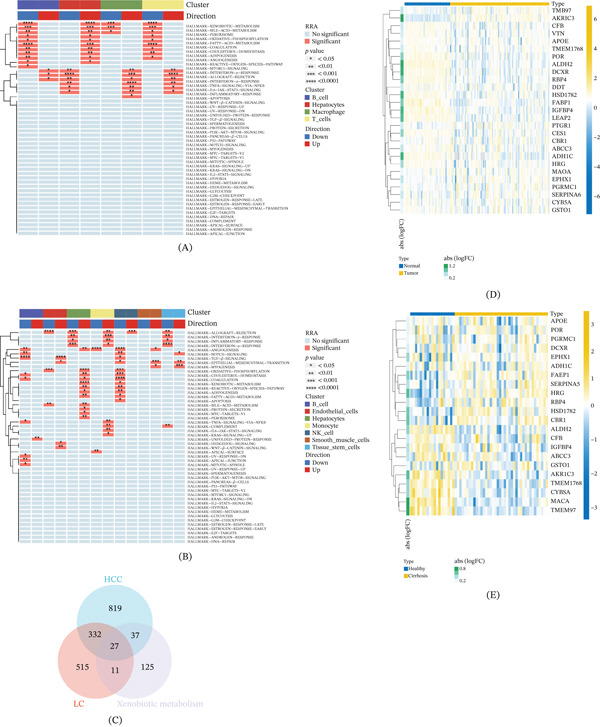
Identification of xenobiotic metabolism‐related genes dysregulated in both HCC and LC. (A) Heatmap of classical carcinogenic pathway analysis of HCC samples. (B) Heatmap of classical carcinogenic pathway analysis of LC samples. (C) Venn diagram showing the intersection of xenobiotic metabolism‐related hallmark genes (from MSigDB), HCC marker genes, and LC marker genes. In this study, “HCC marker genes” and “LC marker genes” refer to differentially expressed genes (DEGs) identified from bulk transcriptome data by comparing HCC or LC samples versus normal samples (|log2FC| > 1, adjusted *p* < 0.05). (D) Heatmap of intersection genes expression levels in HCC bulk transcriptome data. (E) Heatmap of intersection genes expression levels in LC bulk transcriptome data. ∗*p* < 0.05, ∗∗*p* < 0.01, and ∗∗∗*p* < 0.001.

Classical pathway analysis in HCC and LC tissues indicated that the HALLMARK–XENOBIOTIC−METABOLISM pathway was enormously correlated with the cluster of hepatocytes and immune cells in Figure 3A–B. Totally 27 genes were obtained from the intersection of xenobiotic metabolism‐related hallmark genes, HCC marker genes, and LC marker genes (Figure 3C). The expression levels of intersection genes in HCC and LC bulk transcriptome data were visualized in Figure 3D–E.

### 3.3. PPI Network and Function Enrichment Analysis

**Figure 4:**
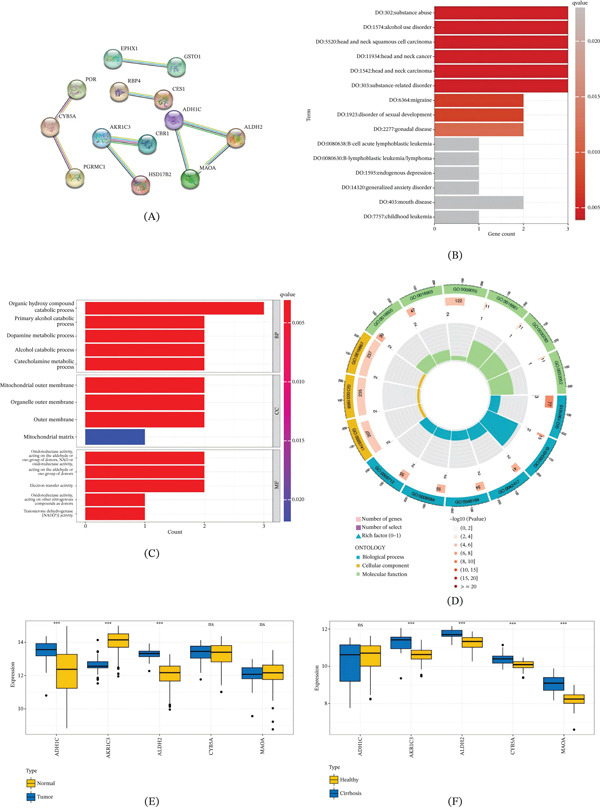
Validation of hub genes in HCC and LC tissues. (A) PPI network of hub genes. (B) Bar diagram of DO enrichment analysis based on hub genes. (C) Bubble map of GO enrichment analysis based on hub genes. (D) Circle diagram of GO enrichment analysis based on hub genes. (E) Expression levels of ADH1C, ALDH2, MAOA, AKR1C3, and CYB5A in HCC bulk transcriptome data. (F) Boxplot of expression levels of ADH1C, ALDH2, MAOA, AKR1C3, and CYB5A in LC bulk transcriptome data. ns, not significant; ∗∗∗*p* < 0.001.

The PPI network was constructed based on the hallmark genes and hub genes were identified, including AKR1C3, CYB5A, ADH1C, MAOA, and ALDH2 (Figure 4A). ADH1C, MAOA, and ALDH2 had close relations and interactions, whereas CYB5A and AKR1C3 were bound up with other genes. We performed GO and DO enrichment analyses to investigate the functional annotation of above genes. The DO terms showed hub genes were enriched in substance abuse, alcohol use disorder, head and neck squamous cell carcinoma, head and neck cancer, head and neck carcinoma, and substance‐related disorder (Figure 4B). The annotation results of the GO analysis exhibited in Figure 4C–D. Enriched Top 5 biological processes are organic hydroxy compound catabolic process, primary alcohol catabolic process, dopamine metabolic process, alcohol catabolic process, and catecholamine metabolic process. These enrichment terms strengthened that hub genes were appropriately annotated and reliable for marker screening. Hub genes expression profiles in HCC (Figure 4E) and LC (Figure 4E) bulk transcriptome data were compared. There was a significant difference in the expression levels of ADH1C, AKR1C3, and ALDH2 in HCC groups compared with the normal groups. The significant difference in the expression levels of CYB5A, MAOA, AKR1C3, and ALDH2 was identified between the cirrhosis and healthy groups.

### 3.4. Diagnostic Value, Prognostic Potential, and Cellular Localization of Hub Genes

First, we evaluated the diagnostic value of the hub genes using ROC curve analysis. The area under the ROC curve (AUC) of ADH1C, AKR1C3, and ALDH2 was 0.781, 0.916, and 0.966, respectively, in the HCC cohort, whereas AUC of AKR1C3, ALDH2, CYB5A, and MAOA was 0.869, 0.911, 0.899, and 0.982, respectively, in the LC cohort (Figure 5A–B). These results indicate that these hub genes possess high sensitivity and specificity as diagnostic biomarkers for distinguishing HCC or LC patients from healthy individuals. UMAP plot in Figure 5C–D highlighted gene expression level per cell for five hub genes. The expression levels of hub genes in various cell types were visualized in Figure 5E,F. In Figure 5E, AKR1C3, CYB5A, MAOA, and ALDH2 exist extensively in hepatocytes and macrophages in HCC cohort. In Figure 5FF, all hub genes are widely expressed in hepatocytes in LC cohort, whereas different expression levels of hub genes are identified in other celltypes. Besides, ALDH2 widely distribute in smooth muscle cells, stem cells, and monocytes, whereas CYB5A in smooth muscle cells, stem cells, and endothelial cells. AKR1C3 is also highly expressed in NK cells.

Figure 5Validation of five hub genes (ADH1C, ALDH2, MAOA, AKR1C3, and CYB5A) in HCC and LC tissues. (A) ROC curve of five hub genes in HCC cohort. (B) ROC curve of five hub genes in LC cohort. (C) UMAP of the distribution of five hub genes in HCC scRNA‐seq data. (D) Umap of the distribution of five hub genes in LC scRNA‐seq data. (E) Violin diagram of expression levels of hub genes in each cell population of HCC scRNA‐seq data. (F) Violin diagram of expression levels of hub genes in each cell population of LC scRNA‐seq data.

**Figure:**
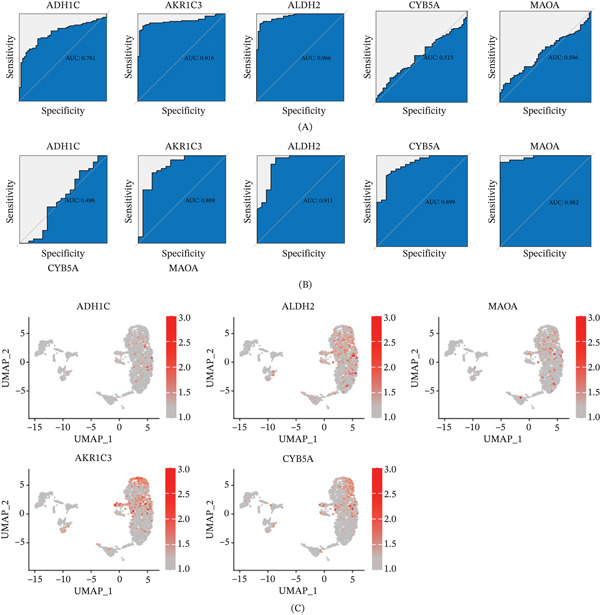
(A)

**Figure:**
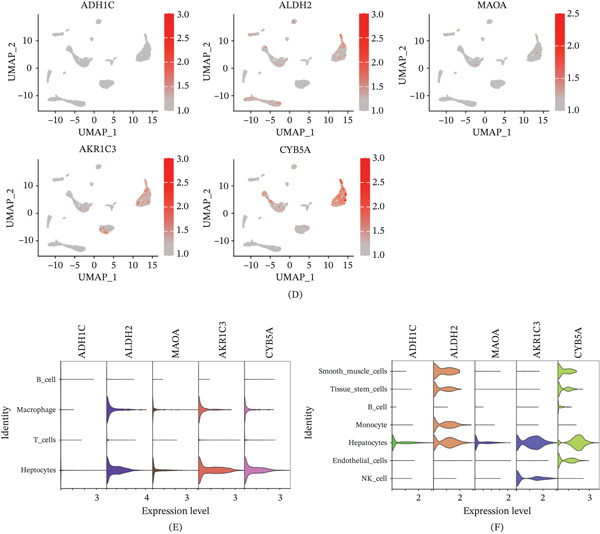
(B)

### 3.5. Correlation of Hub Genes With Immune Cells

Based on the correlation analysis between hub genes, the results exhibite that both ADH1C and MAOA have significantly positive correlationships with ALDH2 and CYB5A in HCC cohort, respectively (Figure 6A–B). Correlation analysis of hub genes with immune cells was identified in HCC and LC cohort (Figure 6C–D). After FDR correction for multiple testing (Benjamini–Hochberg method), the following correlations remained statistically significant (adjusted *p* < 0.05). As shown in Figure 6E, in the HCC cohort, ALDH2 was negatively associated with CD8+ T cells (adjusted *p* < 0.01) and plasma cells (adjusted *p* < 0.05), whereas positively associated with resting CD4+ memory T cells and naive B cells (adjusted *p* < 0.05). CYB5A and MAOA were negatively correlated with CD8+ T cells (adjusted *p* < 0.05).

Figure 6Correlation analysis of five hub genes in HCC and LC cohort. (A) Pie chart of hub gene correlation analysis in HCC cohort. (B) Circle diagram of hub gene correlation analysis in HCC cohort. (C) Pie chart of hub gene correlation analysis in LC cohort. (D) Circle diagram of hub gene correlation analysis in LC cohort. (E) Heatmap of correlation between hub gene and immune cells in HCC cohort. (F) Heatmap of correlation between hub gene and immune cells in LC cohort. ∗*p* < 0.05, ∗∗*p* < 0.01, and ∗∗∗*p* < 0.001.

**Figure:**
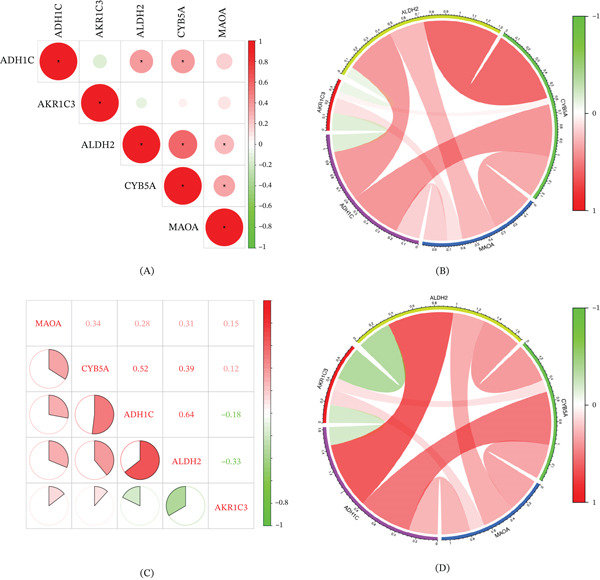
(A)

**Figure:**
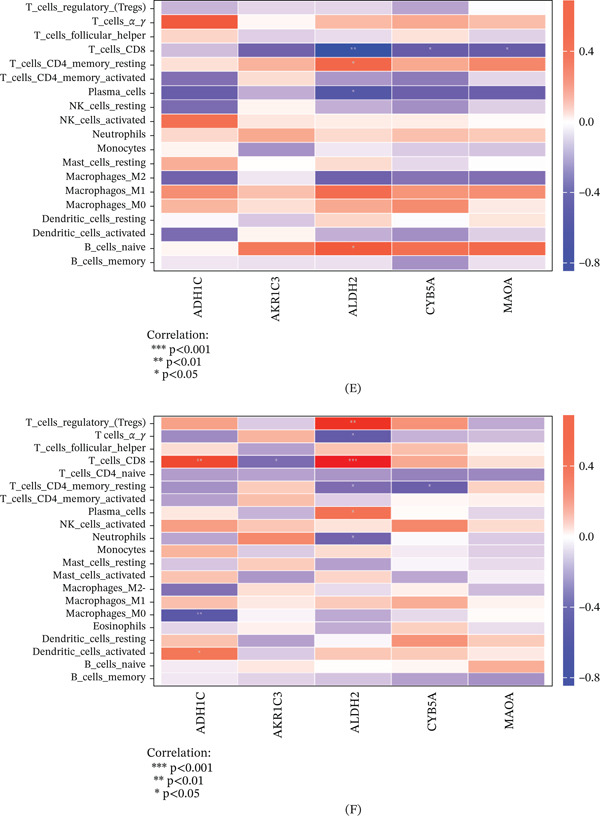
(B)

In the LC cohort, after FDR correction, ALDH2 had a significantly positive correlation with CD8+ T cells (adjusted *p* < 0.001), regulatory T cells (adjusted *p* < 0.01), and plasma cells (adjusted *p* < 0.05), whereas a negative correlation with gamma delta T cells and resting CD4+ memory T cells (adjusted *p* < 0.05). ADH1C had a significantly positive correlation with CD8+ T cells (adjusted *p* < 0.01) and activated dendritic cells (adjusted *p* < 0.05), whereas a negative correlation with M0 macrophages (adjusted *p* < 0.01). A negative correlation between AKR1C3 and CD8+ T cells (adjusted *p* < 0.05) and a negative correlation between CYB5A and resting CD4+ memory T cells (adjusted *p* < 0.05) were identified in the LC cohort (Figure 6F).

### 3.6. Potential Therapeutic Compounds Prediction Based on Hub Genes

**Figure 7:**
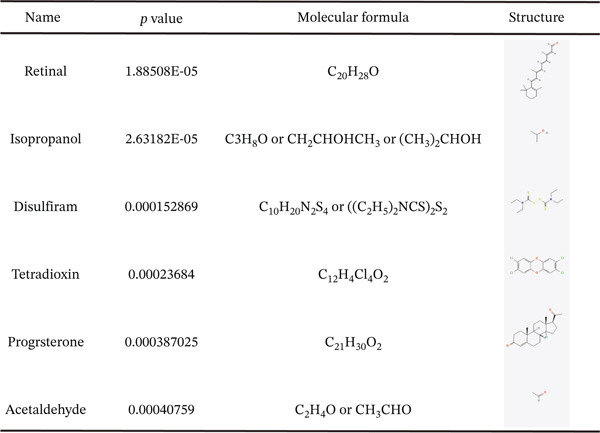
Molecular formulas and two‐dimensional structures of the top six potential therapeutic compounds or pathway modulators identified for HCC and LC based on hub gene signature.

Potential compound prediction based on the hub gene signature identified six bioactive molecules with known interactions to our gene set, including retinal, isopropanol, disulfiram, tetradioxin, progesterone, and acetaldehyde (Figure 7). The enrichment of molecules involved in alcohol metabolism (e.g., isopropanol and acetaldehyde) strongly corroborates the central role of our hub genes in this critical pathway.

### 3.7. Metabolism Hub Genes in HCC and LC: Identification and Functional Verification

This study successfully identified five key hub genes related to xenobiotic metabolism, namely AKR1C3, CYB5A, ADH1C, MAOA, and ALDH2, through bioinformatics analysis. Further differential expression analysis showed that the expression levels of these five hub genes were significantly higher in HCC tissues and LC tissues than in normal liver tissues, with statistical significance (*p* < 0.05). Functional association analysis indicated that the above five genes were closely related to biological processes such as xenobiotic metabolism, substance abuse, alcohol use disorder, and cancer development, suggesting that they may play an important regulatory role in liver tissue lesions, especially the malignant progression of HCC.

**Figure 8:**
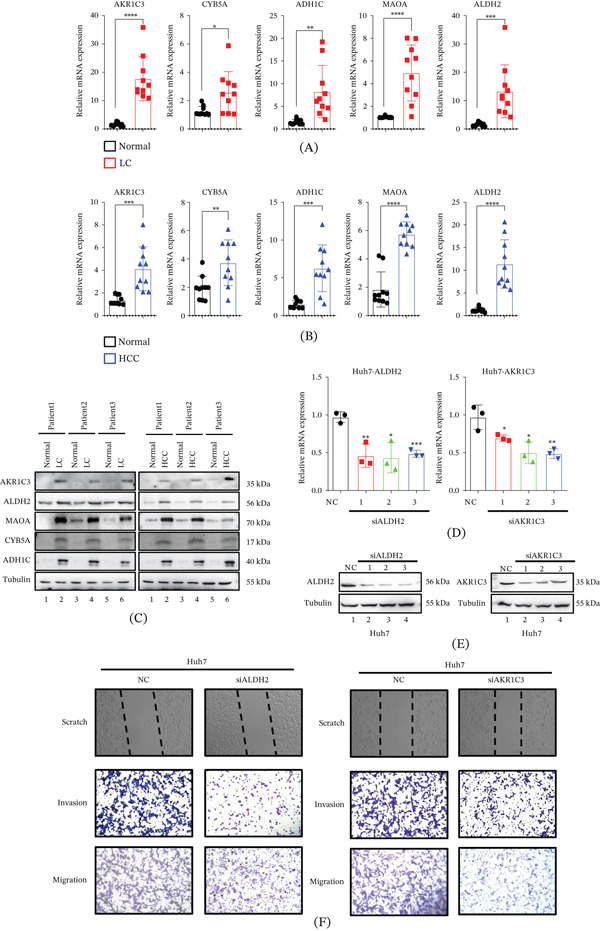
Metabolism hub genes in HCC and LC: identification and functional verification. (A‐B) mRNA expression levels of AKR1C3, CYB5A, ADH1C, MAOA, and ALDH2 in HCC tissues, LC tissues, and corresponding normal tissues were detected by qRT‐PCR. (C) Protein expression levels of the five hub genes in HCC tissues, LC tissues, and normal liver tissues were verified by Western blot analysis. All experiments were performed using at least 10 pairs of HCC tumor/adjacent normal tissues and LC lesion/normal liver tissues. (D–E) The knockdown efficiency of ALDH2 and AKR1C3 in Huh7 cells transfected with specific siRNAs was evaluated by qRT‐PCR and Western blot. (F) The effects of ALDH2 or AKR1C3 knockdown on the migration and invasion abilities of Huh7 cells were assessed by cell scratch assay, transwell migration assay, and transwell invasion assay.∗*p* < 0.05, ∗∗*p* < 0.01, and ∗∗∗*p* < 0.001.

To verify the reliability of the bioinformatics screening results, this study conducted clinical tissue verification experiments, collecting ≥ 10 pairs of HCC tumor tissues and paired adjacent normal tissues, as well as LC lesions and corresponding normal liver tissues under the same criteria. qRT‐PCR confirmed that the mRNA expression levels of the five hub genes in LC (Figure 8A) and HCC (Figure 8B) tissues were significantly higher than those in normal tissues, and Western Blot further verified that their protein expression levels were synchronously increased (Figure 8C). Subsequently, ALDH2 and AKR1C3 were selected for siRNA knockdown in Huh7 human hepatoma cells. qRT‐PCR (Figure 8D) and Western Blot (Figure 8E) confirmed that the knockdown efficiency was more than 70%; cell scratch and transwell migration and invasion experiments showed that after knockdown of the two genes, the migration and invasion abilities of Huh7 cells were significantly decreased, and the number of transmembrane cells was reduced by more than 50% compared with the control group (Figure 8F) (*p* < 0.05). In conclusion, the five hub genes are abnormally highly expressed in HCC and LC tissues, and ALDH2 and AKR1C3 can promote the migration and invasion of hepatoma cells, providing an experimental basis for subsequent studies.

## 4. Discussion

Unlike earlier studies that focused on xenobiotic enzymes in isolation or within a single disease context [14, 15], our approach bridges molecular changes in both LC and HCC. We deliberately analyzed LC and HCC in parallel based on the established “cirrhosis‐HCC axis,” where LC serves as the major precursor for HCC development. This design is pivotal for identifying biomarkers relevant not only for diagnosis of established cancer but also for risk stratification in patients with premalignant cirrhotic disease. The consistent dysregulation of these hub genes across both bulk and single‐cell platforms in LC and HCC suggests their potential involvement in the fibrotic‐to‐malignant transition.

Through integrated transcriptomic analysis, we identified five xenobiotic metabolism‐related hub genes—AKR1C3, CYB5A, ADH1C, MAOA, and ALDH2—with significant diagnostic value in LC and HCC. Functional enrichment analysis revealed that these genes are primarily involved in organic hydroxy compound catabolism, alcohol catabolism, and catecholamine metabolism, processes closely linked to the liver′s detoxification functions. DO enrichment further associated these genes with substance abuse and alcohol use disorders, reinforcing their critical role in liver pathophysiology.

Based on our findings and established literature, we propose several mechanistic hypotheses for how these hub genes may contribute to HCC progression within the LC–HCC continuum. First, their role in detoxification suggests that dysregulation may lead to the accumulation of genotoxic metabolites (e.g., acetaldehyde from impaired ADH1C/ALDH2 activity) that drive DNA damage and mutagenesis. Second, beyond detoxification, these genes influence key signaling pathways [17]; AKR1C3 modulates steroid hormone metabolism, potentially altering estrogen receptor signaling linked to HCC proliferation [18], whereas MAOA regulates serotonin and dopamine levels that may activate profibrotic pathways via neurotransmitter receptors [19]. Third, byproducts of xenobiotic metabolism (e.g., reactive oxygen species from CYB5A‐involved cytochrome P450 cycles) can create a pro‐oxidant microenvironment fostering chronic inflammation and oncogenic transformation [20].

To experimentally validate our bioinformatics findings, we examined the five hub genes in clinical tissue samples and performed functional assays. qRT‐PCR and Western blot confirmed significant upregulation of all five genes at both mRNA and protein levels in HCC and LC tissues compared with normal controls (Figure 8A–C). Given their high diagnostic AUC values and established roles in alcohol metabolism, we selected ALDH2 and AKR1C3 for functional characterization. siRNA‐mediated knockdown of either gene in Huh7 hepatoma cells significantly impaired cell migration and invasion, with > 50% reduction in transwell assays (Figure 8D–F) (*p* < 0.05). These results provide direct evidence that ALDH2 and AKR1C3 promote hepatoma cell motility and invasiveness, supporting their active participation in HCC progression beyond being passive biomarkers.

We also explored the relationship between hub genes and the tumor immune microenvironment. Immune tolerance is a key characteristic of HCC, with complex interactions among infiltrating immune cells and suppressive signaling [21, 22]. In our analysis, ALDH2 and CYB5A showed significant correlations with CD8+ T cells, regulatory T cells, and other immune populations, with distinct patterns between HCC and LC cohorts. These findings suggest that xenobiotic metabolism genes may influence immune cell infiltration and function, though the underlying mechanisms require further investigation.

Additionally, we employed the hub gene signature to predict potentially relevant bioactive compounds. The prediction identified six molecules, including retinal, isopropanol, disulfiram, and acetaldehyde. The strong association with alcohol metabolism‐related molecules computationally validates the pivotal role of our hub genes in this pathway. Notably, acetaldehyde—a known carcinogen—is the substrate detoxified by ALDH2, reinforcing ALDH2 as a compelling therapeutic target. Disulfiram, an FDA‐approved ALDH2 inhibitor, has shown antitumor effects in various cancers [23], making it a particularly interesting candidate for repurposing in HCC. Retinal and progesterone implicate retinoic acid and hormonal signaling pathways, both with established roles in liver pathophysiology [24].

Several limitations should be acknowledged. Our study relies heavily on bioinformatic analyses, and while we provided experimental validation for expression and selected functional assays, direct evidence from in vivo animal models and more comprehensive mechanistic studies are still needed. The sample size for clinical validation was limited, and immune correlation analyses require further confirmation through experimental approaches such as coculture models. Additionally, the diagnostic performance of this gene signature has not been validated in independent prospective cohorts. Future work should focus on elucidating the functional mechanisms of these genes in hepatocytes and stellate cells, their regulation of relevant signaling pathways, and their interactions with immune cells to facilitate clinical translation.

## 5. Conclusion

This study identified five xenobiotic metabolism‐related hub genes (AKR1C3, CYB5A, ADH1C, MAOA, and ALDH2) that were significantly overexpressed in HCC and LC tissues with high diagnostic value. Clinical validation confirmed their upregulation, and functional assays demonstrated that ALDH2 and AKR1C3 promote hepatoma cell migration and invasion. These findings provide new insights into the role of xenobiotic metabolism genes in the cirrhosis‐to‐HCC progression and lay a foundation for future studies.

## Author Contributions

**Hao Xu:** visualization, software, formal analysis, writing – original draft. **Yanpeng Li:** methodology, investigation, funding acquisition, writing – original draft. **Rui Zhao:** visualization, software, data curation, writing – original draft. **Shaowei Ma:** project administration, investigation, writing – original draft. **Ning Hu:** conceptualization, resources, writing – review & editing. **Shuo Yu:** supervision, resources, writing – review & editing. **Xuefeng Lv:** conceptualization, resources, validation, supervision, writing – review & editing. **Hao Xu** and **Yanpeng Li** contributed equally to this work.

## Funding

This study was supported by the Medical Science Research Project of Hebei (20230362).

## Ethics Statement

Our study is not involved in the use of human subjects and animals. No ethical approval and informed consents are required.

## Consent

This study was approved by the Ethics Committee of the Second Hospital of Hebei Medical University (Approval No. 2025‐R172). Informed consent was obtained from all participating patients.

## Conflicts of Interest

The authors declare no conflicts of interest.

## Supporting information


**Supporting Information** Additional supporting information can be found online in the Supporting Information section. Table S1: The sequences of primers for RT‐qPCR.

## Data Availability

The data that support the findings of this study are available from the corresponding authors upon reasonable request.
