# Supplementary material for: Integrated Bulk and Single‐Cell Transcriptomic Analysis Reveals Xenobiotic Metabolism Genes Drive Progression From Liver Cirrhosis to Hepatocellular Carcinoma
Source: Hum Mutat. 2026 May 13;2026:6845605. doi: 10.1155/humu/6845605 (PMC13169128; doi:10.1155/humu/6845605)
Supplement: Supplementary file 1 — Supporting Information Additional supporting information can be found online in the Supporting Information section. Table S1: The sequences of primers for RT‐qPCR. [file HUMU-2026-6845605-s001.docx]

**Table S1. The sequences of primers for RT-qPCR**

|  | Forward (5’-3’) | Reverse (5’-3’) |
| --- | --- | --- |
| β-actin | TGGCACCCAGCACAATGAA | CTAAGTCATAGTCCGCCTAGAAGCA |
| AKR1C3 | GGGATCTCAACGAGACAAACG | AAAGGACTGGGTCCTCCAAGA |
| AMACR | AGTTCTACGAGCTGCTGATCAAA | TGCCTTCGTCTTCTCTGCAAATA |
| ADH1C | TCTGAAAACTCGCCTTCCTCC | CCTGTCGCCTTCGCTCAACTA |
| CYB5A | GAGCATCCTGGTGGAGAAGA | TCTCGTGCATCCGTAGAGTG |
| MAOA | TTCAGGACTATCTGCTGCCAA | GGTCCCACATAAGCTCCACC |
